# Defining Care Patterns and Outcomes Among Persons Living with HIV in Washington, DC: Linkage of Clinical Cohort and Surveillance Data

**DOI:** 10.2196/publichealth.9221

**Published:** 2018-03-16

**Authors:** Amanda D Castel, Arpi Terzian, Jenevieve Opoku, Lindsey Powers Happ, Naji Younes, Michael Kharfen, Alan Greenberg, DC Cohort Executive Committee

**Affiliations:** ^1^ Department of Epidemiology and Biostatistics Milken Institute School of Public Health The George Washington University Washington, DC United States; ^2^ HIV/AIDS, Hepatitis, STD, and TB Administration The District of Columbia Department of Health Washington, DC United States

**Keywords:** HIV/AIDS, health information technology, surveillance, retention, viral suppression, antiretroviral therapy

## Abstract

**Background:**

Triangulation of data from multiple sources such as clinical cohort and surveillance data can help improve our ability to describe care patterns, service utilization, comorbidities, and ultimately measure and monitor clinical outcomes among persons living with HIV infection.

**Objectives:**

The objective of this study was to determine whether linkage of clinical cohort data and routinely collected HIV surveillance data would enhance the completeness and accuracy of each database and improve the understanding of care patterns and clinical outcomes.

**Methods:**

We linked data from the District of Columbia (DC) Cohort, a large HIV observational clinical cohort, with Washington, DC, Department of Health (DOH) surveillance data between January 2011 and June 2015. We determined percent concordance between select variables in the pre- and postlinked databases using kappa test statistics. We compared retention in care (RIC), viral suppression (VS), sexually transmitted diseases (STDs), and non-HIV comorbid conditions (eg, hypertension) and compared HIV clinic visit patterns determined using the prelinked database (DC Cohort) versus the postlinked database (DC Cohort + DOH) using chi-square testing. Additionally, we compared sociodemographic characteristics, RIC, and VS among participants receiving HIV care at ≥3 sites versus <3 sites using chi-square testing.

**Results:**

Of the 6054 DC Cohort participants, 5521 (91.19%) were included in the postlinked database and enrolled at a single DC Cohort site. The majority of the participants was male, black, and had men who have sex with men (MSM) as their HIV risk factor. In the postlinked database, 619 STD diagnoses previously unknown to the DC Cohort were identified. Additionally, the proportion of participants with RIC was higher compared with the prelinked database (59.83%, 2678/4476 vs 64.95%, 2907/4476; *P*<.001) and the proportion with VS was lower (87.85%, 2277/2592 vs 85.15%, 2391/2808; *P*<.001). Almost a quarter of participants (23.06%, 1279/5521) were identified as receiving HIV care at ≥2 sites (postlinked database). The participants using ≥3 care sites were more likely to achieve RIC (80.7%, 234/290 vs 62.61%, 2197/3509) but less likely to achieve VS (72.3%, 154/213 vs 89.51%, 1869/2088). The participants using ≥3 care sites were more likely to have unstable housing (15.1%, 64/424 vs 8.96%, 380/4242), public insurance (86.1%, 365/424 vs 57.57%, 2442/4242), comorbid conditions (eg, hypertension) (37.7%, 160/424 vs 22.98%, 975/4242), and have acquired immunodeficiency syndrome (77.8%, 330/424 vs 61.20%, 2596/4242) (all *P*<.001).

**Conclusions:**

Linking surveillance and clinical data resulted in the improved completeness of each database and a larger volume of available data to evaluate HIV outcomes, allowing for refinement of HIV care continuum estimates. The postlinked database also highlighted important differences between participants who sought HIV care at multiple clinical sites. Our findings suggest that combined datasets can enhance evaluation of HIV-related outcomes across an entire metropolitan area. Future research will evaluate how to best utilize this information to improve outcomes in addition to monitoring them.

## Introduction

A central feature of the updated 2020 National HIV/AIDS (human immunodeficiency virus/acquired immunodeficiency syndrome) Strategy is to measure progress along the HIV care continuum to ensure that target goals are met for each stage. The ability to monitor progress in meeting these goals is often hampered by varying methodologies for data collection, analyses, and variation in measurement approaches, with estimates often relying on either clinic-level or population-based data [1,2]. Both approaches have their advantages and disadvantages. Clinic data provides more detailed and real-time data from the site where care is being delivered, and whether the patient kept or missed primary care visits. However, compared with surveillance-based data, clinic data is less informative for tracking patients who become incarcerated, move, or transfer care [3-5]. These silent transfers of care and the limitation that clinic-attending populations may not represent the general population, present a challenge when trying to make robust estimates of HIV care [6-8]. In contrast, surveillance data are useful for monitoring population-based outcomes but sometimes lack data accuracy and completeness for describing patient-level characteristics and often are subject to reporting time lags [9-16].

In the absence of a unified health record for HIV infected persons, triangulating data from multiple sources such as clinical cohort and surveillance data can help improve our ability to describe care patterns, service utilization, comorbidities and ultimately measure and monitor clinical outcomes. For example, collaborations between local HIV clinics and health departments seeking to identify out-of-care HIV infected patients have found that their combined efforts resulted in timelier, more accurate and complete data, and improved ascertainment of care status [4,8,17,18].

The District of Columbia (DC) Cohort study is a prospective observational clinical cohort study of persons living with HIV/AIDS and receiving care across 13 clinical care sites in Washington, DC [19]. Through an innovative data linkage process, DC Cohort participant data, including sociodemographics, HIV-related diagnosis and laboratory values, and sexually transmitted disease (STD) diagnosis data, are matched with the DC Department of Health (DOH) surveillance data every 6 months [19]. After recognizing the limitations of each database alone, the linkage process was designed to improve the completeness and accuracy of both databases. The primary objectives of this analysis were to perform an assessment of the utility of the linkage process in its ability to improve the completeness of the DC Cohort database and the DOH data. We sought to do this by (1) quantifying the differences between the pre-and postlinked databases, (2) evaluating HIV care continuum outcomes, STD diagnoses, and HIV clinic visit patterns using the prelinked databases compared with the postlinked database, and (3) using the postlinked database to compare sociodemographic characteristics and HIV care continuum outcomes among participants receiving HIV care at multiple sites.

## Methods

### Data Sources

#### The DC Cohort Study

Washington, DC has one of the highest HIV rates among cities in the United States, with 2.0% of its population living with HIV—about 14,000 residents as of 2015 [20]. The design of the DC Cohort study, which began enrollment in 2011, has been described previously [19,21,22]. Its source population consists of adults and children diagnosed with HIV infection who received outpatient HIV care at one or more DC Cohort sites and consented to participate. Participants can consent to participate at multiple clinics in which they receive HIV care. DC Cohort sites include 8 hospital-based or affiliated sites and 5 community-based clinics that collectively serve over half of persons living with HIV/AIDS (PLWHA) in DC [19,20]. Clinical data recorded during HIV care visits were abstracted from each site’s electronic medical record and merged into a centralized Web-based database (Discovere; Cerner Corporation, Kansas City, MO) that collects data on demographics, diagnoses, laboratory tests, pathology and clinical procedures, medications, and drug resistance information. Informed consent included participant acknowledgment of record linkage between patient data collected by DC Cohort study sites and data reported to DC DOH. The study protocol, consent forms, and research instruments were approved by the George Washington University Institutional Review Board (IRB), the DC DOH IRB, and individual study sites’ IRBs [20].

#### DC Department of Health HIV/AIDS Hepatitis, STD, Tuberculosis Administration

The DC DOH has conducted confidential name-based HIV reporting since 2007 and HIV-related electronic laboratory data reporting of cluster of differentiation 4 (CD4) counts and viral load (VL) values since 2009 (22 District of Columbia Municipal Regulations § 206, 21, 23). STD reporting is also conducted in a confidential named-based manner, with over 45,000 syphilis, gonorrhea, and chlamydia cases being reported annually [20]. The HIV/AIDS, Hepatitis, STD, Tuberculosis Administration (HAHSTA) receives over 140,000 HIV- and STD-related laboratory reports from 29 different laboratories annually (HAHSTA internal communication).

### Linkage of DC Cohort and DC Department of Health HIV/AIDS Hepatitis, STD, Tuberculosis Administration Data

#### Linkage Methods

**Figure 1:**
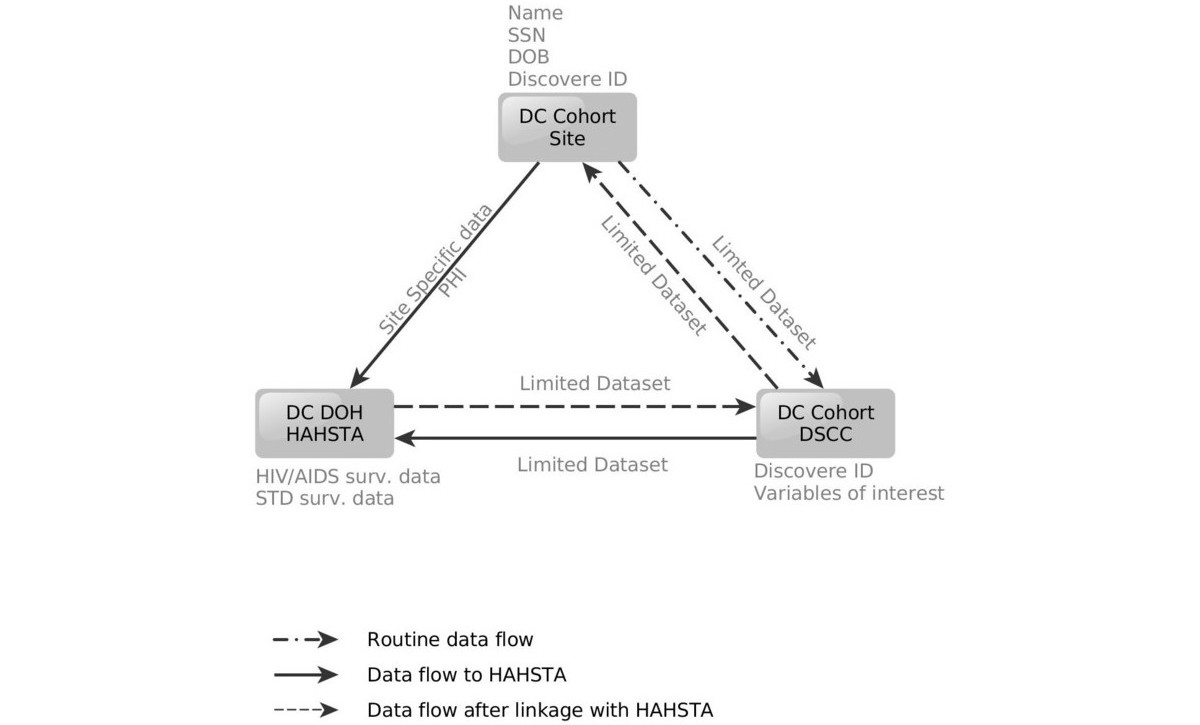
Linkage algorithm for DC Cohort and DC Department of Health (DOH) data. DC DOH HAHSTA: DC Department of Health HIV/AIDS, Hepatitis, STD, TB Administration; SSN: social security number; DOB: date of birth; PHI: personal health information; Surv.: surveillance; DSCC: Data and Statistics Coordinating Center. Variables of interest include those variables that overlap between what is routinely collected in both the DC Cohort and the DC DOH HAHSTA, including but not limited to dates of HIV diagnoses, CD4 (cluster of differentiation 4) and viral laboratory data, opportunistic infections, and sexually transmitted disease diagnoses.

Linkage of the DC Cohort and DC DOH databases is performed semiannually and is ongoing. Data on DC Cohort patients enrolled between January 1, 2011 and June 15, 2015 were linked to this analysis. The linkage algorithm is shown in Figure 1. First, each DC Cohort site sends a limited dataset electronically via a secure file transfer protocol (FTP) site to the DC DOH. The limited dataset includes the study ID, patient name, date of birth, and social security number, if available. Simultaneously, the DC Cohort Data and Statistics Coordinating Center (DSCC) prepares a limited dataset for the DC DOH containing the study identification (ID) as well as HIV-related variables collected at the site. The DOH is authorized to receive both these files since it is already authorized to receive named data on all persons living with HIV/AIDS diagnosed with or receiving HIV care in DC. Additionally, DC Cohort participants provided consent for the linkage [9,23].

Data from the sites and the DSCC are then merged with data from the DC enhanced HIV/AIDS Reporting System (eHARS) and the STD surveillance database (STD*MIS). The postlinkage database containing only the DC Cohort ID is sent back to the DSCC through the FTP site.

#### Linkage Algorithm

Electronic linkage of HIV-related datasets is conducted using an 11-key algorithm, using identifiers including patient first and last name, date of birth, sex at birth and social security number. For both the DC Cohort prelinked and DOH datasets, the algorithm creates identifier-based keys that generated variables to systematically match records in the datasets, while taking into account the misspellings of names and data entry errors. After these 11 variables are created in both datasets, each key is matched separately, producing 11 discrete datasets that were later merged and deduplicated by a patients’ study and eHARS ID. Similarly, DC Cohort and STD surveillance data are matched using a 10-key algorithm, based on identifiers such as first name, last name, date of birth, and sex at birth. After linkage, the combined dataset is deduplicated by study ID, disease type, and disease date.

#### Postlinkage Database

Results from the match (the postlinked database) include data on HIV, AIDS, and STD diagnoses, AIDS-defining opportunistic infections (OIs), laboratory data such as CD4 counts and VL, and vital status. Differences in laboratory dates or laboratory values by the data source (DC Cohort vs DC DOH) are reconciled using fuzzy matching. For the date of HIV or AIDS diagnosis, the earlier date is used regardless of the data source.

The United States Centers for Disease Control and Prevention surveillance guidelines regarding the hierarchical risk of HIV transmission are used to reconcile differences in documented transmission risk, independent of data source [24,25].

### Eligibility Criteria

For this analysis, participants’ data were matched if they were actively enrolled in the DC Cohort as of January 1, 2011, had not withdrawn from the study, or transferred care to another clinical site. To assess continuum of care measures such as retention in care, we reviewed viral load, CD4 tests, and encounters for those participants with at least 1 year of follow-up for the period of June 15, 2014 to June 15, 2015. Participants were considered lost-to-follow-up if, after manual review, no laboratory data from either the DOH or the DC Cohort, and no medical-chart based data from the DC Cohort data were available for 18 months or longer as of June 15, 2015, as per study protocol.

### Receipt of Care by Number of Clinical Sites

To determine the number of clinical sites where a participant was receiving care, CD4 and VL test results, proxies for HIV care, were flagged as originating from either a DC Cohort site or a non-DC Cohort site [26,27]. Receipt of care was grouped into three categories: care at one, two, or three or more sites. Care at one site included participants who only had labs from their DC Cohort enrollment site. Care at two sites included participants enrolled at only one DC Cohort site but who had labs from another site (ie, either a non-DC Cohort site or a site that was not their enrollment site). Care at three or more sites included participants enrolled at only one DC Cohort who had 2 or more labs from two or more other sites (ie, either a non-DC Cohort site or another DC Cohort site that was not their enrollment site). Of note, additional labs obtained through the linkage may have been related to HIV primary care or the result of referrals to specialists who were also drawing HIV-related labs. Since we were unable to determine the reason for the CD4 and VL tests conducted outside of the DC Cohort sites, receipt of care at more than one site does not necessarily indicate receipt of HIV primary care at more than one site.

### HIV Care Continuum Outcomes: Retention in Care on Antiretroviral Therapy and Viral Suppression

A participant was defined as meeting the definition of being retained in care (RIC) if there was evidence of at least two HIV-related encounters (eg, either HIV-related medical visit and/or laboratory test results) at least 90 days apart in a 12-month period from June 15, 2014 to June 15, 2015 [6,16,28-31]. For the purposes of this analysis, a participant was considered RIC even if the encounters occurred at multiple sites. Being on antiretroviral therapy (ART) was defined as being prescribed an ART regimen anytime during the study period, that is, from June 15, 2014 to June 15, 2015. ART status was based solely on prelinked data as ART data are not collected by the DC DOH. Viral suppression (VS) was defined as participants whose last VL on file was <200 copies/mL among those who were retained in care and on ART.

### Statistical Analysis

Frequencies on demographic and clinical characteristics at study enrollment (baseline) were computed in the prelinked DC Cohort database, prelinked DC DOH database, and postlinked database. Chi-square test statistics and Wilcoxon rank-sum tests were used to determine differences among categorical and continuous variables, respectively. Percent concordance between select variables in the prelinked DC Cohort database and postlinked databases were computed using kappa test statistics to assess the comparative accuracy of the databases. Participant outcomes (ie, RIC and VS) in the prelinked DC Cohort database and postlinked database were compared. In the postlinked database, participant demographic and clinical data were also compared based on the number of sites where a participant had evidence of receiving care (1 site, 2 sites, ≥3 sites). These comparisons were made using chi-square test statistics. Statistical comparisons with *P* values <.05 were considered statistically significant. Analyses were conducted in SAS 9.3 (SAS Institute, Inc., Cary, NC) and R (version 3.2.4).

## Results

### Assessment of Differences in Demographic and Clinical Characteristics Between the Pre- and Postlinkage Databases

**Figure 2:**
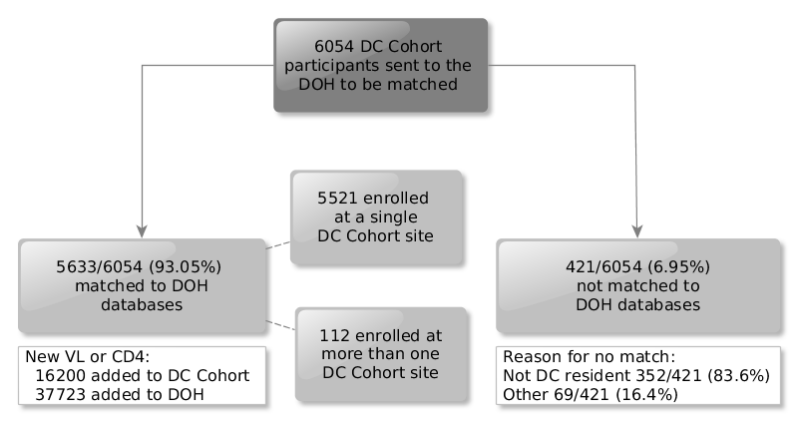
Results of linkage of DC Cohort and DC Department of Health (DOH) surveillance data as of June 2015 (N=5521). DOH: Department of Health; VL: viral load; CD4: cluster of differentiation 4.

The DC Cohort DSCC submitted data on 6054 study IDs to the DC DOH of which 5633/5064 (93.05%) unique participants matched to the DC DOH database and 421/6054 (6.95%) did not (see Figure 2). Of those who did not match, 352/421 (83.6%) were non-DC residents. Among those that matched, 5521/5633 (98.01%) were enrolled at a single DC Cohort site; 112/5521 (2.03%) were enrolled at more than one DC Cohort site. Of the matched participants, 4476/5521 (81.07%) were actively enrolled in the study with at least 1 year of follow-up by the end of 2015.

The demographic and clinical characteristics are displayed by the database from which they were calculated: the prelinked DC Cohort database, the prelinked DC DOH database, and the postlinked database as shown in Table 1. In the prelinked DC Cohort database, among the 5521 participants enrolled at one DC Cohort site, 25.99% (1435/5521) of the study sample was female (data not shown), 4069/5521 (73.70%) non-Hispanic black, and 4093/5521 (74.14%) were DC residents. Nearly 50% (2220/4477) were identified as men who have sex with men (MSM) as their HIV transmission risk. Mean age was 44 years (data not shown) and mean time since HIV diagnosis was 14 years. Since enrollment, 4719/5333 (88.49%) participants had ever been virally suppressed, 521/2273 (22.92%) had an OI at AIDS diagnosis, and 2123/5521 (38.45%) had ever had an STD diagnosis.

When comparing the prelinked DC Cohort database with the postlinked database, a significantly higher percentage of participants were found to be black, deceased, infected through MSM sexual contact, to have had an OI at AIDS diagnosis, and to have ever been virally suppressed. (*P*<.001 for all). The mean duration of HIV diagnosis in the postlinked database increased from 14 to 14.8 years, indicative of earlier diagnosis dates. Additionally, the number of STD diagnoses increased from 2123 to 2739. Furthermore, post linkage, a higher percentage of participants were Maryland and Virginia residents, more infections were attributed to MSM sexual contact and fewer to MSM/IDU, the mean duration of infection increased from 12.2 to 14.8 years, and the proportion of participants ever virally suppressed increased (*P*<.001 for all).

Interrater reliability of selected variables that overlapped between the prelinked DC Cohort database and the postlinked database varied in agreement. There was strong agreement for race/ethnicity (.75) and state of residence (.72); moderate agreement for vital status (κ=.55) and OI at AIDS diagnosis (κ=.40), and poor to fair agreement for transmission risk (κ=.36) and whether a participant had ever been virally suppressed (ie, <200 copies/mL; κ=.20).

The prelinked DC Cohort database included laboratory data collected at clinical sites, while the prelinked DC DOH database included laboratory data collected for surveillance purposes. While the number of CD4 results were fairly similar when comparing prelinked DC Cohort (n=33,505) and prelinked DC DOH (n=35,990) databases, the number of VL results was not. The prelinked DC Cohort database had 31,715 VL results, yet the prelinked DC DOH database had only 12,381 VL results.

### Differences in Demographic and Clinical Characteristics by Number of HIV Care Sites

Differences in demographic and clinical characteristics of DC Cohort participants who were matched through June 15, 2015 were assessed based on the number of HIV care sites using the postlinked database. The number of sites where a participant received HIV care was determined using the source of HIV labs. Of the sample, 4242/5521 (76.83%) had evidence of receiving HIV care at only one DC Cohort site, 855/5521 (15.49%) at two sites, and 424/5521 (7.68%) at three or more sites (Table 2). Those who received care at three or more sites differed demographically and clinically from those who received care at fewer sites; they were more likely to be non-Hispanic black, have a history of AIDS, be homeless or report temporary housing, and to have been referred to substance use treatment. Those receiving care at three or more sites were also more likely to have public insurance, be enrolled in primary care at their DC Cohort site, and receive care at a community-based DC Cohort site. This group also fared worse clinically; they were more likely to have lower CD4 counts (≤350 cells/mm^3^), have a detectable VL (ie, >200 copies/mL), and have uncontrolled viremia (ie, VL ≥100,000 copies/mL) on their most recent VL test. They were also more likely to suffer from comorbid conditions, including hypertension, cardiovascular disease, and mental health issues (*P*<.001; Table 2) and more likely to have died by June 2015.

**Table 1:** Demographic and clinical characteristics of matched participants by data source (DC Cohort and DC Department of Health [DOH]) and linkage status as of June 2015 (N=5521).

Characteristic^a^			Prelinked DC cohort data, n (%)	Prelinked DOH^b^ data, n (%)	Postlinked data, n (%)	Prelinked DC cohort versus postlinked (*P* value^c^)	Prelinked DOH versus postlinked (*P* value)	Agreement/concordance between prelinked DC cohort and postlinked data (Kappa)
**Race/ethnicity**								
		Non-Hispanic black	4069 (73.70)	4142 (75.02)	4175 (75.62)	*<.001*	.01	
		Non-Hispanic white	865 (15.67)	860 (15.58)	912 (16.52)			.75
		Other/unknown^d^	587 (10.63)	519 (9.40)	434 (7.86)			
**State of residence**								
	District of Columbia		4093 (74.14)	4088 (74.04)	4091 (74.10)	>.99	*<.001*	
	Maryland		1040 (18.84)	882 (15.98)	1042 (18.87)			.72
	Virginia		314 (5.69)	269 (4.87)	313 (5.65)			
	Other		74 (1.43)	282 (5.11)	75 (1.36)			
Vital status			106 (1.92)	122 (2.21)	163 (2.95)	*<.001*	.016	.55
**Transmission risk^e^**								
	MSM^f^/IDU^g^		75 (1.68)	206 (4.31)	93 (1.74)	*<.001*	*<.001*	
	MSM		2220 (49.59)	2311 (48.36)	2850 (53.23)			
	Heterosexual		1519 (33.93)	1374 (28.75)	1439 (26.88)			.36
	Perinatal		223 (4.98)		215 (4.02)			
	Other		440 (9.83)	888 (18.58)	757 (14.14)			
	Mean HIV duration in years (IQR^h^)		14.0 (8.3)	12.2 (7.0)	14.8 (8.2)	*<.001*	*<.001*	-
	OI^i^ at AIDS diagnosis^j^		521 (22.92)	975 (30.20)	981 (28.58)	*<.001*	.16	.40
	Ever STD^k^		2123 (38.45)	694 (12.57	2739 (49.61)	-	-	-
	Number of Viral Load labs		31,715	12,381	37,663	-	-	-
	Number of CD4^l^ labs		33,505	35,990	43,757	-	-	-
	Ever virally suppressed^m^		4719 (88.47)	2532 (47.48)	4848 (90.91)	*<.001*	*<.001*	.20

^a^Date of birth and sex at birth were treated as matching variables for the linkage.

^b^DOH: Department of Health.

^c^*P* values for categorical variables were calculated using chi-square tests; *P* values for continuous distributions were obtained from Wilcoxon rank-sum tests. *P* values in italics denote statistical significance at the .001 level.

^d^Other race groups include those of multiple race group and unknown.

^e^The denominator for transmission risk was 4477, 4779, and 5354 for prelinked DC Cohort data, prelinked DOH data and the postlinked data, respectively.

^f^MSM: men who have sex with men.

^g^IDU: male or female injection drug user.

^h^IQR: interquartile range.

^i^OI: opportunistic infection.

^j^Opportunistic infections at AIDS diagnosis is an AIDS-defining condition that does not include those with CD4 counts <200 cells/mm^3^ or CD4% <14. The denominator for OIs was 2273, 3229, and 3433 for prelinked DC Cohort data, prelinked DOH data, and postlinked data, respectively.

^k^STD: sexually transmitted disease.

^l^CD4: cluster of differentiation 4.

^m^The denominator for ever virally suppressed was 5521, 5333, and 5333 for prelinked DC Cohort data, prelinked DOH data, and postlinked data, respectively. Any viral load <200 copies/mL since enrollment was considered suppressed among participants enrolled anytime between January 1, 2011 and June 15, 2015.

**Table 2:** Demographic and clinical characteristics of DC Cohort participants by number of sites where they were receiving HIV-related care (N=5521).

Characteristic		Total^a^	Care at 1 site	Care at 2 sites	Care at ≥3 sites	*P* value^b^
Participants, n (%)		5521 (100.00)	4242 (76.8)	855 (15.49)	424 (7.68)	Not applicable
Mean age at entry (SD)		44.3 (13.2)	44.4 (13.4)	44.1 (912.9)	44.6 (11.4)	.63
**Gender at birth, n (%)**						
	Female	1435 (25.99)	1011 (23.83)	280 (32.7)	144 (34.0)	
**Race/ethnicity, n (%)**						*<.001*
	Non-Hispanic black	4175 (75.62)	3060 (72.14)	726 (84.9)	389 (91.7)	
	Non-Hispanic white	912 (16.52)	805 (18.98)	82 (9.6)	25 (5.9)	
	Other/unknown^c^	434 (7.86)	377 (8.89)	47 (5.5)	10 (2.4)	
**State of residence, n (%)**						*<.001*
	District of Columbia	4091 (74.09)	2915 (68.72)	770 (90.1)	406 (95.8)	
	Maryland	1042 (18.87)	958 (22.58)	69 (8.1)	15 (3.5)	
	Virginia	313 (5.67)	300 (7.07)	13 (1.5)	0 (0)	
	Other	75 (1.36)	69 (1.63)	3 (0.4)	3 (0.7)	
Vital status (died), n (%)		163 (2.95)	110 (2.59)	31 (3.6)	22 (5.2)	*.001*
**Transmission risk^d^, n (%)**						*<.001*
	MSM^e^/IDU^f^	93 (1.74)	58 (1.42)	20 (2.4)	15 (3.6)	
	MSM	2850 (53.23)	2293 (55.95)	386 (46.1)	171 (40.8)	
	Heterosexual	1439 (26.88)	1089 (26.57)	243 (29.0)	107 (25.5)	
	Perinatal	215 (4.02)	170 (4.15)	36 (4.3)	9 (2.1)	
	Other/unknown^g^	757 (14.14)	488 (11.91)	152 (18.2)	117 (27.9)	
**Housing status, n (%)**						*<.001*
	Permanent	4421 (80.07)	3445 (81.21)	663 (77.5)	313 (73.8)	
	Temporary	484 (8.77)	333 (7.85)	96 (11.2)	55 (13.0)	
	Homeless	66 (1.19)	47 (1.11)	10 (1.2)	9 (2.1)	
	Other/unknown	550 (9.96)	417 (9.83)	86 (10.1)	47 (11.1)	
**Employment status, n (%)**						*<.001*
	Working, full-time	1498 (27.13)	1320 (31.12)	150 (17.5)	28 (6.6)	
	Working, part-time	185 (3.35)	145 (3.42)	28 (3.3)	12 (2.8)	
	Unemployed	1373 (24.87)	940 (22.16)	270 (31.6)	163 (38.4)	
	Other^h^	2465 (44.65)	1837 (43.31)	407 (47.6)	221 (52.1)	
**Insurance status, n (%)**						*<.001*
	Private	1697 (30.74)	1487 (35.05)	178 (20.8)	32 (7.5)	
	Public	422 (61.98)	2442 (57.57)	615 (71.9)	365 (86.1)	
	Other	402 (7.28)	313 (7.38)	62 (7.3)	27 (6.4)	
Referral to drug treatment, n (%)		570 (10.32)	375 (8.84)	113 (13.2)	82 (19.3)	*<.001*
Ever AIDS, n (%)		3497 (63.34)	2596 (61.20)	571 (66.8)	330 (77.8)	*<.001*
Mean nadir CD4^i^ cells/mm^3^ (SD)		330.7 (591.6)	329.2 (551.7)	335 (626.2)	336.9 (850.2)	*.02*
**Most recent CD4 cells/mm, n (%)^j^**						*<.001*
	<50	51 (1.21)	25 (0.79)	10 (1.4)	16 (4.5)	
	50-200	282 (6.69)	170 (5.37)	65 (9.4)	47 (13.2)	
	200-350	492 (11.67)	357 (11.27)	82 (11.8)	53 (14.9)	
	350-500	791 (18.76)	602 (19.00)	114 (16.5)	75 (21.1)	
	500+	2601 (61.68)	2014 (63.57)	422 (60.9)	165 (46.3)	
**Most recent viral load copies/ml, n (%)^k^**						*<.001*
	<200	3458 (83.83)	2729 (86.72)	518 (78.5)	211 (66.4)	
	200-1000	188 (4.56)	128 (4.07)	40 (6.1)	20 (6.3)	
	1000-10,000	183 (4.44)	120 (3.81)	39 (5.9)	24 (7.5)	
	10,000-50,000	182 (4.41)	104 (3.30)	40 (6.1)	38 (11.9)	
	50,000-100,000	66 (1.60)	39 (1.24)	14 (2.1)	13 (4.1)	
	100,000+	48 (1.16)	27 (0.86)	9 (1.4)	12 (3.8)	
Primary care at DC Cohort site, n (%)		3790 (68.6)	2774 (65.39)	646 (75.6)	370 (87.3)	*<.001*
**Comorbid conditions, n (%)**						
	Mental health	2345 (42.47)	1650 (38.90)	435 (50.9)	260 (61.3)	*<.001*
	Hypertension	1396 (25.29)	975 (22.98)	261 (30.5)	160 (37.7)	*<.001*
	Cardiovascular	939 (17.01)	662 (15.61)	168 (19.6)	109 (25.7)	*<.001*
	Hepatitis C	586 (10.61)	377 (8.89)	127 (14.9)	82 (19.3)	*<.001*
	Diabetes	554 (10.03)	376 (8.86)	115 (13.5)	63 (14.9)	*<.001*
	Respiratory	473 (8.57)	269 (6.34)	115 (13.5)	89 (21)	*<.001*
	Chronic renal failure	462 (8.37)	310 (7.31)	94 (11.0)	58 (13.7)	*<.001*
	Hepatitis B	136 (2.46)	107 (2.52)	7 (0.8)	22 (5.2)	.20
	Chronic liver disease	139 (2.52)	106 (2.50)	20 (2.3)	13 (3.1)	.66
**Type of clinic, n (%)**						*<.001*
	Hospital-based	3024 (54.77)	2563 (60.42)	365 (42.7)	96 (22.6)	
	Community-based	2497 (45.23)	1679 (39.58)	490 (57.3)	328 (77.4)	

^a^Data are for participants enrolled through June 15, 2015. Care at one site included singly-enrolled participants who had 0 or ≥1 lab from their DC Cohort enrollment site. Care at two sites included singly-enrolled participants who had 0 or ≥1 lab from their DC Cohort enrollment site and ≥1 lab from a second site (ie, a non-DC Cohort site or another DC Cohort site that was not their enrollment site). Care at three or more sites included singly-enrolled participants who had 0 or ≥1 lab from their DC Cohort site and ≥2 labs from ≥2 other sites (ie, a non-DC Cohort site or another DC Cohort site that was not their enrollment site).

^b^*P* values for categorical variables were calculated using chi-square tests; *P* values for continuous distributions were obtained from Wilcoxon rank-sum tests. *P* values in italics denote statistical significance at the .001 level.

^c^Other race groups include those with multiple races and missing; unknown is unknown race/ethnicity.

^d^The denominator for transmission risk was 5354, 4098, 837, and 419 for the total sample, care at 1 site, care at 2 sites, and care at ≥3 sites, respectively.

^e^MSM: men who have sex with men.

^f^IDU: male or female injection drug user.

^g^Other transmission risk includes missing and risk not identified.

^h^Other employment status includes student, disabled, retired and other/unknown.

^i^CD4: cluster of differentiation 4.

^j^The denominators for CD4 count are 4217, 3168, 693, and 356 for the total sample, care at 1 site, care at 2 sites, and care at ≥3 sites, respectively.

^k^The denominators for viral load are 4125, 3147, 660, and 318 for the total sample, care at 1 site, care at 2 sites, and care at ≥3 sites, respectively.

**Figure 3:**
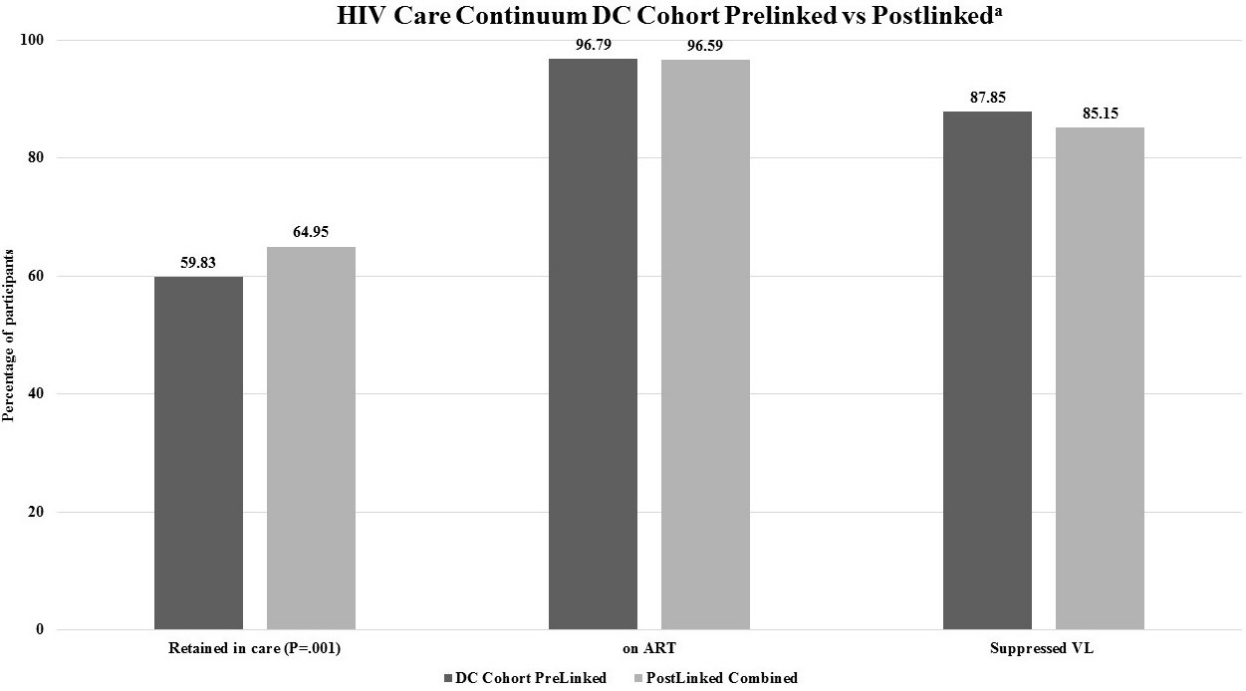
Percentage of participants matched, retained in care, on antiretroviral therapy (ART) and having suppressed viral load (VL) in Washington, DC 2014-2015 stratified by linkage status (pre vs post) (N=4476). The symbol "a" signifies DC Cohort participants who matched with DC Department of Health records and were actively enrolled, not withdrawn, or transferred care from the Cohort, alive, and with at least 1 year of follow-up as of June 15, 2014. Retention in care was defined as matched participants with evidence of at least two HIV-related encounters (eg, either HIV-related medical visit and/or laboratory test results) at least 90 days apart in a 12-month period from June 2014 to June 2015. Being on ART was defined as the number of Cohort participants who were prescribed an antiretroviral therapy (ART) regimen that overlapped with the study period. ART status was based on prelinked data as ART data are not collected by the DC DOH. Suppressed viral load (VL) was defined as matched participants whose last VL was <200 copies/mL among those who were retained in care and on ART.

### Differences in Care Continuum Outcomes

**Figure 4:**
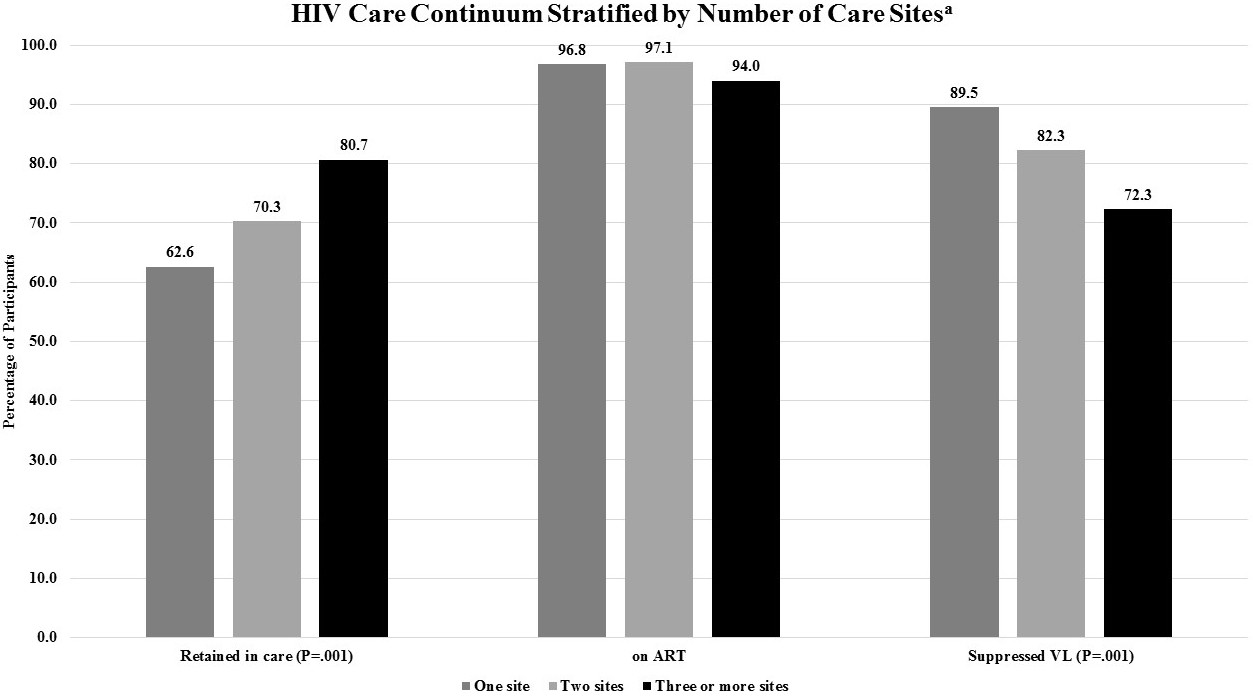
Percentage of participants matched, retained in care, on antiretroviral therapy (ART) and having suppressed viral load (VL) in Washington, DC 2014-2015 stratified by receipt of care at 1, 2 or ≥3 sites (N=4476). The letter "a" signifies DC Cohort participants who matched with DC Department of Health records and were actively enrolled, not withdrawn or transferred care from the Cohort, alive, and with at least 1 year of follow-up as of June 15, 2014. Retention in care was defined as matched participants with evidence of at least two HIV-related encounters (eg, either HIV-related medical visit and/or laboratory test results) at least 90 days apart in a 12-month period from June 2014 to June 2015. Being on ART was defined as the number of Cohort participants who were prescribed an antiretroviral therapy (ART) regimen that overlapped with the study period. ART status was based on prelinked data as ART data are not collected by the DC DOH. Suppressed viral load (VL) was defined as matched participants who had a VL test in the time period and whose last VL was <200 copies/mL among those who were retained in care and on ART.

Among the 4476 participants who were actively enrolled in the study with at least 1 year of follow-up as of June 15, 2014, when measuring the care continuum using the prelinked DC Cohort database compared with the postlinked database, we found that retention in care was higher (59.83% (2678/4476) vs 64.95% (2907/4476); however, the proportion with viral suppression was lower (87.85% (2277/2592) vs 85.15% (2391/2808) (*P*<.001 for both) (see Figure 3). The proportion of participants on ART was high at 96.79% (2592/2678), and was only able to be assessed in the prelinked DC Cohort database. In the postlinked database, the proportion of participants classified as retained and as virally suppressed differed according to the number of sites where care was being received (see Figure 4). Those participants who received care at three or more sites were more likely to meet the definition of retention in care (80.7%, 234/290) compared with those receiving care at one site (62.61%, 2197/3509; *P*<.001) but were less likely to be virally suppressed (72.3% (154/213) vs 89.51% (1869/2088); *P*<.001).

## Discussion

### Principal Findings

Linking clinical data collected in an observational HIV cohort with routinely collected public health surveillance data was mutually beneficial for both the clinical database as well as public health surveillance efforts. Specifically, for the DC Cohort, the linkage improved the accuracy of dates of diagnosis, vital status, and modes of transmission and resulted in the identification of more than 600 additional STD diagnoses, which may otherwise have not been captured in the Cohort database. From the DC DOH perspective, linkage of surveillance data to the DC Cohort database found that the majority of participants had been captured in the DC DOH surveillance data (93%) consistent with the relatively high completeness of surveillance reporting [32]. In addition, among those not matching, most were not DC residents, highlighting the large volume of care being delivered to non-DC residents by DC-based clinics. The linkage also improved measurement of dates of diagnoses, modes of transmission for HIV, and viral suppression for both databases.

With respect to the completeness of laboratory reporting, the linkage resulted in a substantial increase in the number of VLs post linkage. Further examination of the VLs included in the prelinked DC DOH surveillance data revealed that very few results were under 200 copies/mL. This is also reflected in the finding that only 47.5% of participants in the prelinked DC DOH database had ever achieved viral suppression. Given that a relatively high proportion of individuals obtaining care in DC have achieved an undetectable VL [33], this likely reflects that although reportable, all VL values may not be as routinely reported to the DC DOH surveillance program, whereas CD4 results are included in surveillance data regardless of the numeric value [34]. Furthermore, because the prelinked DC Cohort data includes non-DC residents using DC health care facilities, VL labs for individuals who live outside of Washington, DC, are reported to their respective health departments and may not be captured in the DC DOH surveillance database. Given the relatively high proportion of non-DC residents participating in the Cohort who did not match to the DC DOH surveillance database, this may further explain the differences in VL reporting. The DC DOH surveillance program is constantly striving to improve the completeness of all laboratory reporting through routine checks with laboratory facilities and standard regional data exchanges. Nevertheless, despite the low initial number of VLs included in the DC DOH database, they were still of added value to the DC Cohort database.

Discriminating between DC Cohort and non-DC Cohort HIV laboratories was also key to ascertaining whether participants were coenrolled at more than one DC Cohort site or receiving care at multiple sites throughout the city, or receiving care at DC Cohort sites and non-DC Cohort sites. While most DC Cohort participants were receiving care at one site, almost one-quarter had evidence of receipt of HIV-related care at two or more sites. Furthermore, participants with evidence of care at three or more sites fared worse clinically and while they were most likely to be retained in care, they were less likely to be virally suppressed. These findings were consistent with previous analyses on-site migration in DC which also found lower CD4 and higher VLs among persons seeking care at more than one site [35]. These trends may be reflective of other individual-level factors such as homelessness, substance abuse, and more fragmented care in general, among patients with multiple comorbid conditions. Furthermore, this more vulnerable group may have had seemingly higher retention in care as they may have returned to care more often for follow-up visits based on provider concerns about client health, fear of losing contact with the most transient clients, or based on receipt of more referrals to other clinics or specialists for their comorbid conditions [35,36]. However, while a higher proportion of these participants may have met the retention in care definition, their care patterns appear to reflect more disparate care, as meeting the definition did not translate into higher viral suppression. Thus, given the complexity in measuring retention in care with the shifting standards of clinical care and movement across clinics, emphasis should be placed on achieving viral suppression—a clear goal of treatment.

Additional laboratory data, used as supplemental and complementary information, allowed for re-estimation of retention and viral suppression and improved understanding of drop-offs along the HIV care continuum. Using a nested care continuum approach in which each step is dependent on the prior step, our initial clinic-based care continuum would have underestimated the percentage of participants meeting the retention in care definition and overestimated viral suppression; however, by combining additional laboratories from the health department, we were able to achieve a more accurate measure of these key indicators. Hence, routine data linkages such as these could assist in refining the accuracy of care continua and help prioritize clinical and public health interventions that seek to re-engage persons who are not optimally in care [37,38].

Overall, our care continuum estimates were similar to those of other HIV cohorts in the United States, including the HIV Outpatient Study (HOPS), a convenience sample of patients at selected HIV clinics in the United States, and the Medical Monitoring Project (MMP) study, a multisite supplemental surveillance system in the United States designed to provide nationally representative data on PLWHA [38]. DC Cohort estimates for proportion ‘on ART’ and viral suppression fell within the range of HOPS and MMP estimates in 2012 (97% and 92% on ART, respectively and 85% and 78% virally suppressed, respectively). DC Cohort estimates were also comparable to findings from the North American AIDS Cohort Collaboration on Research and Design (NA-ACCORD). Among more than 35,000 NA-ACCORD participants with at least one HIV care visit in the first 6 months of 2008, 82% were prescribed ART and 78% had suppressed VLs [39].

### Limitations

This study has certain noteworthy limitations. Without knowing the full context in which CD4 and VL labs were drawn, deriving inference about the ability of DC Cohort participants to establish and maintain a primary medical home for HIV care is a challenge. We cannot exclude the possibility that additional labs may be the result of referrals to specialists who are also drawing HIV-related labs or acute encounters with the medical system such as emergency department visits and hospitalizations. Given the way laboratory results are reported, we are unable to fully describe the source of the laboratory (eg, inpatient, emergency department, outpatient, a specialty of reporting provider, etc), which would allow for better characterization of care pattern by type of encounter. However, additional analyses to determine whether participants were receiving care sequentially at these sites versus in an overlapping manner, may help further delineate these care patterns. Finally, DC Cohort participants may not be generalizable to all HIV-infected persons in DC, given that a certain proportion of PLWH in DC is not consistently engaged in care [30]. In future analyses, we intend to compare characteristics of DC Cohort participants to city-wide HIV population characteristics to assess whether Cohort-based care estimates approximate care trajectories for the city as a whole.

### Conclusions

Despite these limitations, this analysis represents a successful triangulation of data from clinical cohort and public health surveillance data and demonstrated that the data linkages were mutually beneficial. The linkage not only helped to improve the accuracy and completeness of each database but also helped to describe care patterns among PLWHA, and enhanced measurement of clinical outcomes and the HIV care continuum at a population-level. The results derived through combining these databases will help inform HIV programmatic efforts and strengthen the DC DOH surveillance system as they will not only enhance the completeness of case data but contribute to the measurement of a more complete care continuum. The DC Cohort intends to use these data to inform the development of interventions focused on case management and improved care coordination across clinical sites and across jurisdictions. The DC DOH will be retooling its approaches to ensure continuity of care in DC and the surrounding metropolitan area in an enhanced data-to-care intervention strategy. With a more complete and relevant dataset, DC DOH will collaborate with community providers and deploy its public health team to address interruptions in care. DC DOH also has a data sharing agreement and protocol with Maryland and Virginia to ensure that the most complete data available can be used to inform jurisdictional partners in their data-to-care activities. Performance and findings from this type of linkage provide a reference point for design and interpretation of data from similar data linkages in North America and could potentially be used at the regional and national level as we strive to improve care outcomes [28,39,40].

## Abbreviations

ART: antiretroviral therapy
CD4: cluster of differentiation count
DC: District of Columbia
DOH: Department of Health
DSCC: Data and Statistics Coordinating Center
eHARS: enhanced HIV/AIDS Reporting System
FTP: file transfer protocol
HAHSTA: HIV/AIDS, Hepatitis, STD, Tuberculosis Administration
HOPS: HIV Outpatient Study
IDU: injection drug user
ID: identification
IRB: Institutional Review Board
IQR: interquartile range
MMP: Medical Monitoring Project
MSM: men who have sex with men
NA-ACCORD: North American AIDS Cohort Collaboration on Research and Design
OI: opportunistic infection
PLWHA: persons living with HIV or AIDS
RIC: retention in care
STD: sexually transmitted disease
STD*MIS: Sexually Transmitted Diseases Management Information System
VL: viral load
VS: viral suppression
